# When Should Potentially False Research Findings Be Considered Acceptable?

**DOI:** 10.1371/journal.pmed.0040026

**Published:** 2007-02-27

**Authors:** Benjamin Djulbegovic, Iztok Hozo

## Abstract

Ioannidis estimated that most published research findings are false [1], but he did not indicate when, if at all, potentially false research results may be considered as acceptable to society. We combined our two previously published models [2,3] to calculate the probability above which research findings may become acceptable. A new model indicates that the probability above which research results should be accepted depends on the expected payback from the research (the benefits) and the inadvertent consequences (the harms). This probability may dramatically change depending on our willingness to tolerate error in accepting false research findings. Our acceptance of research findings changes as a function of what we call “acceptable regret,” i.e., our tolerance of making a wrong decision in accepting the research hypothesis. We illustrate our findings by providing a new framework for early stopping rules in clinical research (i.e., when should we accept early findings from a clinical trial indicating the benefits as true?). Obtaining absolute “truth” in research is impossible, and so society has to decide when less-than-perfect results may become acceptable.

## Abstract 2

The authors calculate the probability above which potentially false research findings may become acceptable to society.

As society pours more resources into medical research, it will increasingly realize that the research “payback” always represents a mixture of false and true findings. This tradeoff is similar to the tradeoff seen with other societal investments—for example, economic development can lead to environmental harms while measures to increase national security can erode civil liberties. In most of the enterprises that define modern society, we are willing to accept these tradeoffs. In other words, there is a threshold (or likelihood) at which a particular policy becomes socially acceptable.

In the case of medical research, we can similarly try to define a threshold by asking: “When should potentially false research findings become acceptable to society?” In other words, at what probability are research findings determined to be sufficiently true and when should we be willing to accept the results of this research?

## Defining the “Threshold Probability”

As in most investment strategies, our willingness to accept particular research findings will depend on the expected payback (the benefits) and the inadvertent consequences (the harms) of the research. We begin by defining a “positive” finding in research in the same way that Ioannidis defined it [1]. A positive finding occurs when the claim for an alternative hypothesis (instead of the null hypothesis) can be accepted at a particular, pre-specified statistical significance. The probability that a research result is true (the posterior probability; PPV) depends on: (1) the probability of it being true before the study is undertaken (the prior probability), (2) the statistical power of the study, and (3) the statistical significance of the research result. The PPV may also be influenced by bias [1,4], i.e., by systematic misrepresentation of the research due to inadequacies in the design, conduct, or analysis [1].

However, the calculation of PPV tells us nothing about whether a particular research result is acceptable to researchers or not. Nevertheless, it can be shown that there is some probability (the “threshold probability,” p_t_) above which the results of a study will be sufficient for researchers to accept them as “true” [3]. The threshold probability will depend on the ratio of net benefits/harms (B/H) that is generated by the study [3,5,6]. Mathematically the relationship between p_t_ and B/H can be expressed as (see Appendix, Equation A1):


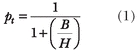


We define net benefit as the difference between the values of the outcomes of the action taken under the research hypothesis and the null hypothesis, respectively (when in fact the research hypothesis is true). Net harms are defined as the difference between the values of the outcomes of the action taken under the null and the research hypotheses, respectively (when in fact the null hypothesis is true) [3]. It follows that if the PPV is above p_t_ we can rationally accept the results of the research findings. Similarly, if the PPV is below p_t_ we should accept the null hypothesis. Note that the research payoffs (the benefits) and the inadvertent consequences (harms) in Equation 1 can be expressed in a variety of units. In clinical research these units would typically be length of life, morbidity or mortality rates, absence of pain, cost, and strength of individual or societal preference for a given outcome [3].

We can now frame the crucial question of interest as: What is the minimum B/H ratio for the given PPV for which the research hypothesis has a greater value than the null hypothesis? Mathematically, this will occur when (see Appendix, Equations A1 and A2):


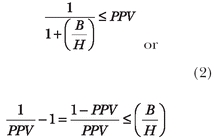


## Calculation of the Threshold Probability of “Accepted Truth”

**Figure 1:**
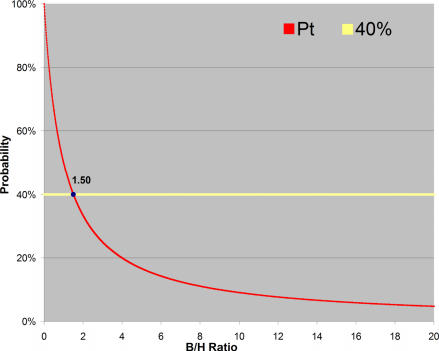
The Threshold Probability **Above** (P_t_ in Red) Which We Should Accept Findings of Research Hypothesis as Being True The horizontal yellow line indicates the actual conditional probability that the research hypothesis is true in the case of positive findings. This means that for benefit/harm ratios above the threshold (1.5 in this example), the research hypothesis can be accepted.

Figure 1 shows the threshold probability of “truth” (i.e., the probability above which the research findings may be accepted) as a function of B/H associated with the research results. The graph shows that as long as the probability of “accepted truth” (a horizontal line) is above the threshold probability curve, the research findings may be accepted. The higher the B/H ratio, the less certain we need to be of the truthfulness of the research results in order to accept them.

Note that we are following the classic decision theory approach to the results of clinical trials, which states that a rational decision maker should select the research versus the null hypothesis depending on which one maximizes the value of consequences [7–9]. In the parlance of expected utility decision theory, this means that we should choose the option with the higher expected utility [3,5,7–12]. (Expected utility is the average of all possible results weighted by their corresponding probabilities—see Appendix). In other words, the results of the research hypothesis should be accepted when the benefit of the action outweigh its harms.

## A Practical Example: When Should We Stop a Clinical Trial?

Interim analyses of clinical trials are challenging exercises in which researchers and/or data safety monitoring committees have to make a judgment as to whether to accept early promising results and terminate a trial or whether the trial should continue [13,14]. If the interim analysis shows significant benefit in efficacy for the new treatment over the standard treatment, continuing to enroll patients into the trial may mean that many patients will receive the inferior standard treatment [13,14]. The first randomized controlled trial of circumcision for preventing heterosexual transmission of HIV, for example, was terminated early after the interim analysis showed that circumcised men were less likely to be infected with HIV [15]. However, if a study is wrongly terminated for presumed benefits, this could result in adoption of a new therapy of questionable efficacy [13,14].

We now illustrate these issues by considering a clinical research hypothesis: is radiotherapy plus chemotherapy (combined R_x_) superior to radiotherapy alone (RT) in the management of cancer of the esophagus? (see Box 1). We consider two scenarios: (1) the best-case scenario (B/H = 13.5), and (2) the worst-case scenario (B/H = 1.4). The probability that the research finding is true [16,17] (i.e., that combined treatment is truly better than radiotherapy alone) under the best-case scenario is 95% [95% confidence interval (CI), 89%–99.9%]. Under the worst-case scenario, the probability that combined treatment is better than radiotherapy alone is 80% [95% CI, 61%–99%]. The threshold probability above which these findings should be accepted is 7% [95% CI, 0%–30%] if we assume that B/H = 13.5, or 41% [95% CI, 11%–72%] if we assume B/H = 1.4 (Table 1).

**Table 1:**
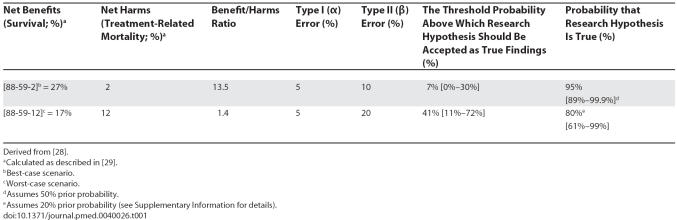
How True Is the Research Hypothesis that Combined Chemotherapy Is Superior To Radiotherapy Alone in the Management of Esophageal Cancer?

**Box 1. Is Combined Chemotherapy Plus Radiotherapy Superior To Radiotherapy Alone for Treating Esophageal Cancer?**

The Radiation Oncology Cooperative Group conducted a randomized controlled trial to evaluate the effects of combined chemotherapy and radiotherapy versus radiotherapy alone in patients with cancer of the esophagus [28].

A sample size of 150 patients was planned to detect an improvement in the two-year survival rate from 10%–30% in favor of combined R_x_ (at α = 0.05 and ß = 0.10). At the interim analysis, 88% of patients in the control group (RT) had died while only 59% in the experimental arm (combined R_x_) had died, resulting in a survival advantage of 29% in favor of combined R_x_ (*p* < 0.001).

For this reason, the trial was terminated prematurely after enrolling 121 patients. Two percent of patients died as a result of treatment in the combined R_x_ group versus 0% in the RT arm. Thus, the observed net benefit/harm ratio in this trial was [88-59-2]/2 = 13.5 [29] (the *best-case scenario*).

For our *worst-case scenario* we assume that two-thirds of patients who experienced life-threatening toxicities with combined R_x_ (12%) will have died. This will result in the worst-case net benefit/harms ratio = (88-59-12)/12 = 1.4.

The trial was stopped using classic inferential statistics which indicated that the probability of the observed results, assuming the null hypothesis that combined R_x_ is equivalent to RT, was extremely small (*p* < 0.001). This, however, tells us nothing about how true the alternative hypothesis is [16,17], i.e., in our case, what is the probability that combined R_x_ is better than RT? The probability that the research finding is true [16,17] (i.e., that combined R_x_ is truly better treatment than RT) under the best-case scenario is 95% [95% CI, 89%–99.9%]. Under the worst-case scenario, the probability that combined R_x_ is better than RT is 80% [95% CI, 61%–99%].

The results indicate that in the best-case scenario, the probability that the research findings are true far exceeds the threshold above which the results should be accepted (i.e., PPV is greater than p_t_). Therefore, rationally, in this case we should not hesitate to accept the findings from this study as truthful. However, in the worst-case scenario, the lower limit of the PPV's 95% confidence interval intersects with the upper limit of the threshold's 95% confidence interval, indicating that under these circumstances the research hypothesis may not be acceptable (since PPV is possibly less than p_t_). Had the investigators made a mistake when they terminated the trial early?

## Dealing with Unavoidable Erroneous Research Findings

Mistakes are an integral part of research. Positive research findings may subsequently be shown to be false [18]. When we accept that our initially positive research findings were in fact false, we may discover that another alternative (i.e., the null hypothesis) would have been preferable [7,19–21]. When an initially positive research finding turns out to be false, this may bring a sense of loss or regret [19,20,22,23]. However, abundant experience has shown that there are many situations in which we can tolerate wrong decisions, and others in which we cannot [2]. We have previously described the concept of *acceptable regret*, i.e., under certain conditions making a wrong decision will not be particularly burdensome to the decision maker [2].

## Defining Tolerable Limits for Accepting Potentially False Results

We now apply the concept of acceptable regret to address the question of whether potentially false research findings should be tolerated. In other words: which decision (regarding a research hypothesis) should we make if we want to ensure that the regret is less than a predetermined (minimal acceptable) regret, R_0_ [2]? (*R_0_* denotes acceptable regret and should be expressed in the same units as benefits and harms).

It can easily be shown that we should be willing to accept the results of potentially false research findings as long as the posterior probability of it being true is above the acceptable regret threshold probability, *p_r_* (see Equation 3, Appendix, and Equations A3 and A4):

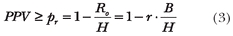
where *r* is the amount of acceptable regret expressed as a percentage of the benefits that we are willing to lose in case our decision proves to be the wrong one (i.e., *R_o_* = *r* · *B*).

This equation describes the effect of acceptable regret on the threshold probability (Equation 1) in such a way that the PPV now also needs to be above the threshold defined in Equation 3 for the research results to become acceptable.

Note that actions under expected utility theory (EUT) and acceptable regret may not necessary be identical, but arguably the most rational course of action would be to select those research findings with the highest expected utility while keeping regret below the acceptable levels. The supplementary material (a longer version of the paper and Appendix) show that the maximum possible fraction of benefits that we can forgo (and still be wrong) while at the same time adhering to the precepts of EUT is given by (see Appendix, Equations A3–A6):


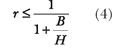


A practical interpretation of this inequality is that some research findings may never become acceptable unless we are ready to violate the axioms of EUT, i.e., accept value r to be larger than defined in Equation 4 (Table 2).

**Table 2:**
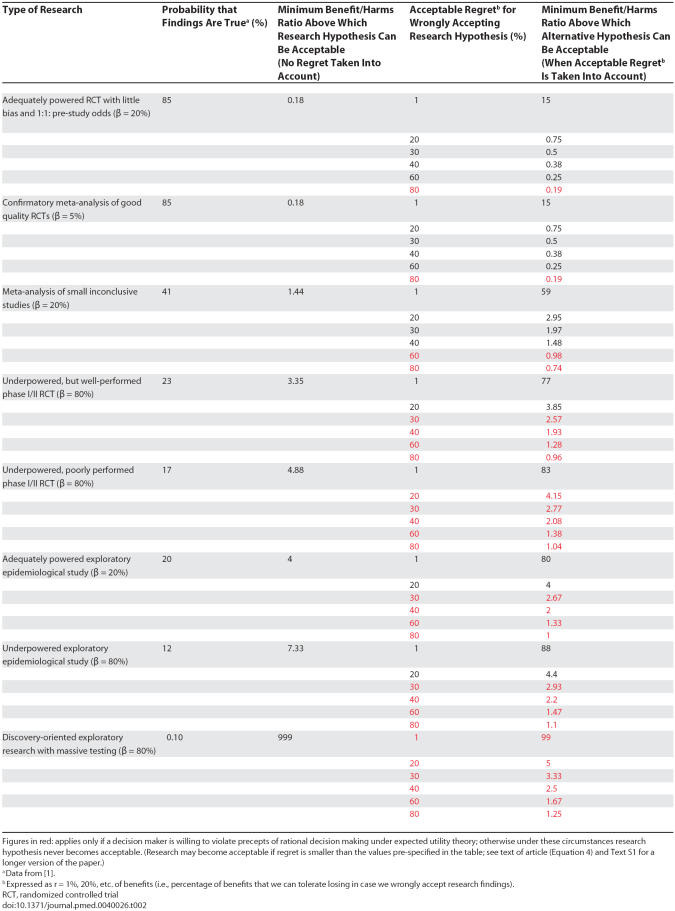
Probability that Research Findings Are True and Benefit/Harms Ratio Above Which Findings May Become Acceptable

We return now to the “real life” scenario above, i.e., the dilemma of whether to stop a clinical trial early. In our worst-case analysis (Box 1), we found that the probability that combined R_x_ is better than radiotherapy alone could potentially be as low as 80% [95% CI, 61%–99%]. This figure overlaps with the probability of the threshold of 41% [95% CI, 11%–72%] above which research findings are acceptable under the worst case scenario (see Table 1) (i.e., PPV is possibly less than p_t_; see Equations 1 and 2). Thus, it is quite conceivable that the investigators made a mistake when they closed the trial prematurely.

One way to handle situations in which evidence is not solidly established is to explicitly take into account the possibility that one can make a mistake and wrongly accept the results of a research hypothesis. Accepting this possibility can, in turn, help us determine “decision thresholds” that will take into account the amount of error which may or may not be particularly troublesome to us if we wrongly accept research findings.

**Figure 2:**
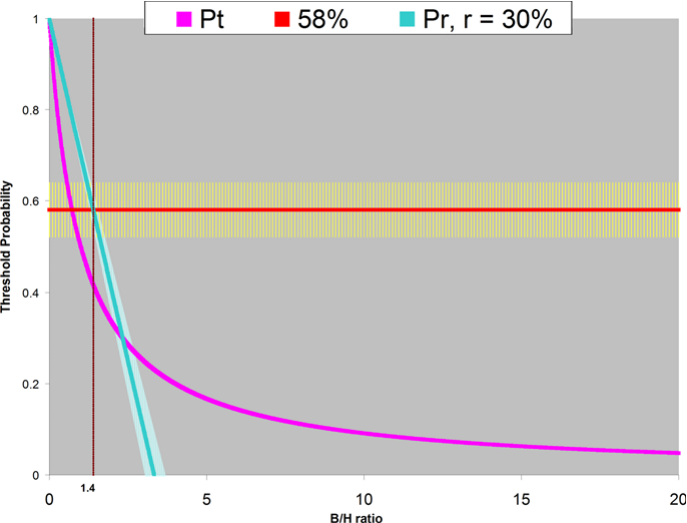
The Threshold Probability (P_t_) **Above** Which We Should Accept Findings of Research Hypothesis as Being True (Pink Line) as a Function of Benefit/Harm Ratio The calculated (acceptable regret) threshold above which we should accept research findings is shown for the worst-case scenario (B/H = 1.4; see text for details) with a (hypothetical) assumption that we are willing to forgo 30% of the benefits (slanted line). The calculated threshold probability (acceptable regret threshold) has a value of 58% when B/H = 1.4 (the horizontal line). This means that as long as the probability that research findings are true is above this acceptable regret threshold, these research findings could be accepted with tolerable amount of regret in case the research hypothesis proves to be wrong (for didactic purposes only one acceptable regret threshold is shown). See Box 2 and text for details.

Let us assume that the investigators in the esophageal cancer trial are prepared to accept that they may be wrong and that they were willing to forgo 10%, 30%, or 67% of benefits. Using Equation 3, the calculations in Box 2 and Figure 2 show that for any willingness to tolerate loss of net benefits of greater than 10%, the probability that combined R_x_ is superior to R_T_ is above all decision thresholds (since p_r_ = 0 in best-case scenario; Equation 3). Therefore the investigators seemed to have been correct when they terminated the trial earlier than originally anticipated.

**Box 2. Determining the Threshold Above Which Research Findings Are Acceptable When Acceptable Regret Is Taken Into Account**

You will recall (in Box 1) that the Radiation Oncology Cooperative Group investigators hoped to detect an absolute difference of 10%–30% in survival in favor of combined R_x_. By finding that combined R_x_ improved survival by 29%, they appeared to have realized their most optimistic expectations [28]. This implies that the investigators would consider their trial a success even if the survival was improved by 10% instead, i.e., less than 67% of the realized, but most optimistic outcome [1-(.10/.30) × 100% = 67%].

Therefore, we assume that the investigators in the esophageal cancer trial are prepared to accept that they may be wrong and that they were willing to forgo 10%, 30%, or 67% of benefits.

We applied Equation 3 to calculate acceptable regret thresholds above which we can accept research findings as true (i.e., when PPV > p_r_).

**Best-case scenario (benefit/harm ratio: 13.5).** The calculated thresholds above which we should accept the findings are zero, regardless of whether our tolerable loss of benefits was 10%, 30%, or 67%. Note that these thresholds (p_r_ = 0) are well below calculated probability that the research hypothesis is true [PPV = 95% (88%–99.9%)] ( i.e., PPV > p_r_ = 0 for all acceptable regret assumptions; Equation 3, Table 1) and hence the research hypothesis should be accepted.

**Worst-case scenario (benefit/harm ratio: 1.4).** The calculated threshold above which we should accept the findings from this study is 86% [95% CI, 84%–88%] for a loss of 10% of benefits, 58% [95% CI, 52%–64%] for a loss of 30% of net benefits, and 6% [95% CI, 0%–19%] if we are willing to tolerate a loss of 67% of net benefits. This means that, except in the case when acceptable regret is 10% or less, the probability that combined R_x_ is superior to RT [80% (61%–99%)] is above all other decision thresholds and its “truthfulness” can be accepted (because PPV [= 80% (61%–99%)] > acceptable regret threshold [= 58% (52%–64%)] and PPV > acceptable regret threshold [= 6% (0%– 19%)]). Note that in case of our willingness to tolerate loss of 30% of benefits for being wrong, the upper limit of the acceptable regret CI (=64%) still overlaps with the lower limit of PPV's CI (=61%), but that is not the case if we are willing to forgo 67% of treatment benefits. See Equation 3, Table 1.

## Threshold Probabilities in Various Types of Clinical Research

Table 2 summarizes the results of most types of clinical research showing the probabilities that the research findings are true and the benefit/harms ratio above which the findings become acceptable. For each type of research, the table shows these probabilities with and without acceptable regret being taken into account. What is remarkable is that depending on the amount of acceptable regret, our acceptance of potentially false research findings may dramatically change. For example, in the case of a meta-analysis of small inconclusive studies, we can accept the research hypothesis as true only if B/H > 1.44. However, if we are willing to forgo, say, only 1% of the net benefits in case we prove to be mistaken, the B/H ratio for accepting the findings from the meta-analysis of small inconclusive studies dramatically increases to 59.

## Conclusion

In the final analysis, the answer to the question posed in the title of this paper, “When should potentially false research findings be considered acceptable?” has much to do with our beliefs about what constitutes knowledge itself [24]. The answer depends on the question of how much we are willing to tolerate the research results being wrong. Equation 3 shows an important result: if we are not willing to accept any possibility that our decision to accept a research finding could be wrong (r = 0), that would mean that we can operate only at absolute certainty in the “truth” of a research hypothesis (i.e., PPV = 100%). This is clearly not an attainable goal [1]. Therefore, our acceptability of “truth” depends on how much we care about being wrong. In our attempts to balance these tradeoffs, the value that we place on benefits, harms, and degrees of errors that we can tolerate becomes crucial.

However, because a typical clinical research hypothesis is formulated to test for benefits, we have here postulated a relationship between *acceptable regret* and the fraction of benefits that we are willing to forgo in the case of false research findings. Unfortunately, when we move outside the realm of medical treatments and interventions, the immediate and long-term harms and benefits are very difficult to quantify. On occasion, wrongly adopting some false positive findings may lead to the adoption of other false findings, thus creating fields replete with spurious claims. One typical example is the use of stem cell transplant for breast cancer, which resulted in tens of thousands of women getting aggressive, toxic, and very expensive treatment based on strong beliefs obtained in early phase I/II trials until controlled, randomized trials demonstrated no benefits but increased harms of stem cell transplants compared with conventional chemotherapy [25]. Therefore, even for clinical medicine, where benefits and harms are more typically measured, we should acknowledge that often the quality of the information on harms is suboptimal [26]. There is no guarantee that the “benefits” will exceed the “harms.” Although (as noted in Text S1) there is nothing to prevent us from relating R_0_ to harms, or both benefits and harms, one must acknowledge that there is much more uncertainty, often total ignorance, about harms (since data on harms is often limited). As a consequence, under these circumstances research may become acceptable only if we relax our criteria for acceptable regret, i.e., accept value r to be larger than defined in Equation 4. In other words, unless we are ready to violate the precepts of rational decision making (see the figures in red in Table 2), a research finding with low PPV (the majority of research findings) should not be accepted [1].

We conclude that since obtaining the absolute “truth” in research is impossible, society has to decide when less-than-perfect results may become acceptable. The approach presented here, advocating that the research hypothesis should be accepted when it is coherent with beliefs “upon which a man is prepared to act” [27], may facilitate decision making in scientific research.

## Supporting Information

Text S1 Longer version of the paper (657 KB DOC).

Text S2 Appendix (163 KB DOC).

## Abbreviations

B/H: net benefits/harms
CI: confidence interval
combined R_x_: radiotherapy plus chemotherapy
EUT: expected utility theory
PPV: posterior probability
p_r_: acceptable regret threshold probability
p_t_: threshold probability
R_0_: acceptable regret
RT: radiotherapy alone

## References

[pmed-0040026-b001] Ioannidis JP (2005). Why most published research findings are false. PLoS Med.

[pmed-0040026-b002] Djulbegovic B, Hozo I, Schwartz A, McMasters K (1999). Acceptable regret in medical decision making. Med Hypotheses.

[pmed-0040026-b003] Djulbegovic B, Hozo I (2002). At what degree of belief in a research hypothesis is a trial in humans justified?. J Eval Clin Practice.

[pmed-0040026-b004] Wacholoder S, Chanock S, Garcia-Closas M, El Ghormli L, Rothman N (2004). Assessing the probability that a positive report is false: An approach for molecular epidemiology studies. J National Cancer Inst.

[pmed-0040026-b005] Pauker S, Kassirer J (1975). Therapeutic decision making: A cost benefit analysis. N Engl J Med.

[pmed-0040026-b006] Djulbegovic B, Desoky AH (1996). Equation and nomogram for calculation of testing and treatment thresholds. Med Decis Making.

[pmed-0040026-b007] Bell DE, Raiffa H, Tversky A (1988). Decision making: Descriptive, normative, and prescriptive interactions.

[pmed-0040026-b008] Hastie R, Dawes RM (2001). Rational choice in an uncertain world.

[pmed-0040026-b009] Ciampi A, Till JE (1980). Null results in clinical trials: The need for a decision-theory approach. Br J Cancer.

[pmed-0040026-b010] Browner WS, Newman TB (1987). Are all significant p values created equal? The analogy between diagnostic tests and clinical research. JAMA.

[pmed-0040026-b011] Hulley SB, Cummings SR (1992). Designing clinical research.

[pmed-0040026-b012] Pater JL, Willan AR (1984). Clinical trials as diagnostic tests. Controlled Clin Trials.

[pmed-0040026-b013] DAMOCLES Study Group (2005). A proposed charter for clinical trial data monitoring committees: Helping them to do their job well. Lancet.

[pmed-0040026-b014] Pocock SJ (2005). When (not) to stop a clinical trial for benefit. JAMA.

[pmed-0040026-b015] Auvert B, Taljaard D, Lagarde E, Sobngwi-Tambekou J, Sitta R (2005). Randomized, controlled intervention trial of male circumcision for reduction of HIV infection risk: The ANRS 1265 trial. PLoS Med.

[pmed-0040026-b016] Goodman SN (1999). Toward evidence-based medical statistics. 1: The p value fallacy. Ann Intern Med.

[pmed-0040026-b017] Goodman SN (1999). Toward evidence-based medical statistics. 2: The Bayes factor. Ann Intern Med.

[pmed-0040026-b018] Ioannidis JPA (2005). Contradicted and initially stronger effects in highly cited clinical research. JAMA.

[pmed-0040026-b019] Bell DE (1982). Regret in decision making under uncertainty. Oper Res.

[pmed-0040026-b020] Loomes G, Sugden R (1982). Regret theory: An alternative theory of rational choice. Economic J.

[pmed-0040026-b021] Loomes G (1987). Testing for regret and disappointment in choice under uncertainty. Economic J.

[pmed-0040026-b022] Allais M (1953). Le compartment de l'homme rationnel devant le risque. Critque des postulates et axiomes de l'ecole Americaine. Econometrica.

[pmed-0040026-b023] Hilden J, Glasziou P (1996). Regret graphs, diagnostic uncertainty and Youden's index. Stat Med.

[pmed-0040026-b024] Ashcroft R (1999). Equipoise, knowledge and ethics in clinical research and practice. Bioethics.

[pmed-0040026-b025] Welch HG, Mogielnicki J (2002). Presumed benefit: Lessons from the American experience with marrow transplantation for breast cancer. BMJ.

[pmed-0040026-b026] Ioannidis JPA, Evans SJW, Gotzsche PC, O'Neill RT, Altman DG (2004). Better reporting of harms in randomized trials: An extension of the CONSORT statement. Ann Intern Med.

[pmed-0040026-b027] deWaal C (2005). On pragmatism.

[pmed-0040026-b028] Herskovic A, Martz K, Al-Sarraf M, Leichman L, Brindle J (1992). Combined chemotherapy and radiotherapy compared with radiotherapy alone in patients with cancer of the esophagus. N Engl J Med.

[pmed-0040026-b029] Djulbegovic B, Hozo I, Lyman G (2000). Linking evidence-based medicine therapeutic summary measures to clinical decision analysis. MedGenMed.

