# Supplementary material for: When Should Potentially False Research Findings Be Considered Acceptable?
Source: PLoS Med. 2007 Feb 27;4(2):e26. doi: 10.1371/journal.pmed.0040026 (PMC1808081; doi:10.1371/journal.pmed.0040026)
Supplement: Text S1 — (657 KB DOC). [file pmed.0040026.sd001.doc]

# When should potentially false research findings be acceptable? Threshold probability and acceptable regret approach

Benjamin Djulbegovic and Iztok Hozo

Department of Interdisciplinary Oncology, H. Lee Moffitt Cancer Center & Research Institute at the University of South Florida

Indiana University Northwest

Department of Mathematics

Gary

Indiana

Funding: Department of Interdisciplinary Oncology, H. Lee Moffitt Cancer Center & Research Institute at the University of South Florida

The funding source has no role in design, analysis, or interpretation of this research study.

Correspondence

Benjamin Djulbegovic, MD,PhD

Professor of Oncology and Medicine

H. Lee Moffitt Cancer Center & Research Institute at the University of South Florida

Department of Interdisciplinary Oncology

12902 Magnolia Drive

Tampa, FL 33612

E-mail: [djulbebm@moffitt.usf.edu](mailto:djulbebm@moffitt.usf.edu)

**Summary**

**Summary**

Recently, Ioannidis (1)estimated that most published research findings are false, but he did not indicate when, if at all, potentially false research results may become acceptable. We combined our two previously published models(2, 3) to calculate the probability above which research findings may become acceptable. A new model indicates that the probability above which research results should be accepted depends on the research’s expected payback (benefits) and inadvertent consequences (harms).This probability may dramatically change depending on our willingness to tolerate error in accepting false research findings. Only in circumstances of insisting on absolute certainty in “truth” of research hypothesis, we will not be willing to accept any possibility that our decision to accept research finding could be wrong. Since this is clearly not attainable goal, we demonstrate how acceptance of research findings changes as a function of acceptable regret i.e. our tolerance toward making a wrong decision of accepting research hypothesis. We illustrate our findings by providing a new framework for early stopping rules in clinical research (i.e. when should we accept early findings from a clinical trial indicating benefits as true).

Since obtaining absolute “truth” in research is impossible, society has to decide when less-than-perfect results may become acceptable.

**Key words**: scientific hypotheses- probability- clinical trials-decision theory

Through the years various commentators have attempted to draw attention to the “scandal of poor medical research” indicating that most published research findings are false(4, 5). More recently, Ioannidis(1) and Wacholder et al(6) provided the quantitative estimate regarding the probability of research findings to be false. Although one may expect this work to help improve a future ratio between true vs. false positive findings in medical research, it will remain impossible that every new research study returns true findings. As society pours its resources in the medical research, it will increasingly realize that the research “payback” always represents a mixture of false and true findings. This is similar to any trade-off in which society invests such as economical development vs. environmental protection, information access vs. confidentiality, security (e.g. eavesdropping of potential criminal activities) vs. civil liberties etc. It is, therefore, evident that with respect to most enterprises that define the modern society, we are willing to accept these trade-offs. In other words, there is some likelihood in which a particular policy becomes socially acceptable. In the case of medical research, the question may be asked “when should potentially false research findings become acceptable?” That is, at which probability are research findings determined to be true and when should we be willing to accept the results of this research?

**Methodological Framework: linking consequences of outcomes of research to the probability above which the results may be considered “true”**

Researchers, physicians, and the public are frequently faced with the problem of deciding when, if at all, they should accept the results of a research study to be sufficiently true in order to allow the findings to be acted upon. As in most investment strategies, our willingness to accept particular research findings will depend on the expected payback (benefits) and inadvertent consequences (harms) of the research. In this paper, we focus on clinical research, but the analysis can be easily extended to other fields. Our approach represents the extension of models that we previously reported(2, 3, 7, 8).

Mathematical details of our methods are provided in the Appendix. Briefly. analogously to Ioannidis(1), we also define “positive” research findings as a claim that alternative hypothesis may be accepted at certain pre-specified statistical significance instead of null findings. In fact, as demonstrated previously, the probability that a research result is true depends on the probability of it being true before the study is undertaken (the prior probability), the statistical power of the study and the statistical significance(1, 6).

The posterior probability that the positive research finding is accurate is given by $PPV=\frac{\left(1-\beta\right)\pi}{\left(1-\beta\right)\pi+\alpha\left(1-\pi\right)}$ where *π* is the prior probability of positive research finding, α is the probability of a false positive research finding (type I error),β the probability of false negative research finding (type II error). (Typically in clinical research α and β are fixed at 0.05 and 0.20, respectively). This classical Bayesian formula was previously reported by others(9-11) and served as a basis for conclusions derived by Ioannidis (1)and Wacholder et al(6). Ioannidis (1) also incorporated the effect of bias in the equation describing PPV. The equation gives us the conditional probability that a research finding is true (or false = *1* - *PPV*) after a positive research result, but does not tell us if this particular result is acceptable or not. Nevertheless, it can be shown that there is some probability above which the results of the study will be sufficient for researchers to accept them as “true”(3). This probability, which we will denote as a threshold probability (p_t_), will depend on the ratio of net benefits/harms (B/H) which is generated in the study(3). Mathematically this can be expressed as:

$p_{t}=\frac{1}{1+\left(\frac{B}{H}\right)}$ (1)

We define net benefit (B) as the difference between the utilities of outcomes of the action taken under research hypothesis and the null hypothesis, respectively (when in fact research hypothesis is true). Net harms (H) are defined as the difference between the utilities of outcomes of the action taken under the null and research hypothesis, respectively (when in fact null hypothesis is true)(3).

The methods outlined in this section follow the classic decision theory approach to the results of clinical trials, which states that a rational decision-maker should select research vs. null-hypothesis depending on which one maximizes the value of consequences(12-14). In parlance of decision-theory, this means that we should choose the alternative with the higher expected utility(3, 12-17). (Expected utility represents an average utility of all possible results weighted by their corresponding probabilities-see Appendix for the details)(2, 3, 12, 14).

According to expected utility decision theory (see Appendix) if *PPV* (the posterior probability that research hypothesis is true) is above *p_t_* we can rationally accept the results of research findings. In other words, the results of research hypothesis should be accepted when benefit of action outweigh its harms.

Similarly, if *PPV* < *p_t_* we should accept the null hypothesis. Note that research payoffs (benefits) and inadvertent consequences (harms) in equation 1) can be expressed in a variety of units. In clinical research these units would typically be length of life, morbidity or mortality rates, absence of pain, cost, strength of individual or societal preference for a given outcome(3). Except for the example which serves to illustrate our method, in this paper net benefits and net harms relates to a generic case of rewards and consequences associated with the research results(3). A reader is referred to other papers for definitions and other practical examples of benefits and harms in clinical medicine(8, 18-21).

Note that in terms of prior probability, *π* (the probability that the research hypothesis is true before we perform the hypothesis testing), this threshold splits into two threshold probabilities: a) the treatment threshold probability above which we will accept the results of research hypothesis (reject a null hypothesis) (*p_tRH_*) and b) the no-treatment threshold probability below which we will accept the results of null hypothesis (*p_tNH_*)(3).

Mathematically these two thresholds can be expressed as:

$p_{t}=\frac{1}{1+LR\cdot\frac{B}{H}}$ (1’)

where *p_t_* = *p_tRH_* if LR=LRneg, and *p_t_* = *p_tNH_* if LR=LRpos. LR (likelihood ratio) can be expressed as LRpos=(1-β)/α and LRneg =β/(1-α) and indicates evidentiary support for research hypothesis(3, 11, 22).Note that the equation is analogous to a general threshold equation which is also applicable to clinical diagnostic and treatment decisions(23). (A reader is referred to our other paper which dealt with the issue at what degree of prior belief in a research hypothesis, the research findings should be considered true(3)).

In this paper, we focus on when the posterior probability that research hypothesis is true can be accepted. The question we are interested in is finding out what is the minimum B/H ratio for the given posterior probability for which the research hypothesis has a greater expected utility than the null hypothesis. This will occur when:

$\frac{1}{1+\left(\frac{B}{H}\right)}\leq PPV$ or $\frac{1}{PPV}-1=\frac{1-PPV}{PPV}\leq\left(\frac{B}{H}\right)$ (2)

We also compute confidence intervals around PPV and threshold estimates to obtain more realistic picture of the “zone” above which research findings should be accepted(7) (see Appendix).

**Calculation of the threshold probability of “accepted truth”**

Figure 1 (equation 1) shows the threshold probability of “truth” (the probability above which research findings may be accepted as true) as a function of benefit/harms ratio associated with the research results. The graph shows that as long as the probability of “accepted truth” (a horizontal line) is above the threshold probability curve the research findings may be accepted. The higher B/H ratio, the less certain we need to be in truthfulness of research results in order to accept them.

**A practical example: when is the research hypothesis that one treatment is better than the other true?**

We illustrate our method by applying it to difficult decisions regarding acceptability of research results while data may still be evolving and the benefit/harm ratio is not well established. This is a situation when we are at a higher risk to make a mistake and erroneously accept the results of research hypothesis, which subsequently may prove to be false. We will illustrate this approach using an example from the clinical research study, which was terminated prematurely for benefits at the interim analyses(24). The interim analyses are challenging exercises in which researchers and/or Data Safety Monitoring Committees have to make judgments whether to accept early promising results and terminate a trial, or to decide whether the trial should continue(25, 26). Continuing enrollment of patients in the light of significant benefit in efficacy for the new treatment may mean that many patients will receive an inferior standard treatments(25, 26). However, if a study is wrongly terminated for presumed benefits, this could result in adoption of a new therapy of questionable efficacy (25, 26).

Our model can help facilitate the decision-making in this situation.

Under auspice of the NCI Radiation Oncology Cooperative Group (RTOG), Herskovic et al. conducted a randomized controlled trial (RCT) to evaluate the effects of combined chemotherapy and radiotherapy (Combined Rx) vs. radiotherapy (RT) alone in patients with cancer of the esophagus(27). A sample size of 150 patients was planned to detect an improvement in the two-year survival from 10-30% in favor of Combined Rx (at α=0.05 and β=0.10). The trial, however, was closed prematurely after enrolling 121 patients when at the interim analysis was determined that 88% of patients in control group vs. 59% in experimental arm had died resulting in survival advantage of 29% in favor of Combined Rx (p<0.001). At the same time, 2% of the patients died as a result of treatment in Combined Rx group vs. 0% in RT arm. Thus, the observed net benefit/harm ratio in this trial was [88-59-2]/2=13.5(19). Although the trial was well done, assume for the purpose of illustration that this was the best-case scenario. Often early optimistic results are not later confirmed due to bias or as a result of chance that may have played a role in the originally conducted clinical research(28, 29) For example, many patients who experienced life-threatening side-effects but survived in research centers of excellence where the original trial was conducted, may have inferior survival when the treatment is exported to the community setting. Therefore, for our worst-case scenario we assume that two-thirds of patients who experienced life-threatening toxicities with Combined Rx (12%) will have died. This will result in the worst-case net benefit/harms ratio = (88-59-12)/12 = 1.4. We also assume that initially strong statistical findings (p<0.001) is now lost and that the study is only borderline significant p=0.05 dropping power to 80%. The trial that was originally terminated had a power of 90% to detect 29% survival difference at α=0.05.

It is important to note that the trial was stopped based on classic inferential statistics which indicated that the probability of the observed results under assumption of the null hypothesis that combined Rx is equivalent to RT was extremely small (p<0.001). This, however, tells us nothing about how true is the alternative hypothesis(11, 22) i.e. in our case what is the probability that Combined Rx is better than RT. Using an approach advocated by Goodman(11, 22), we calculated the probability that research findings are true (i.e. that combined Rx is truly better treatment than RT) was 95% [95%CI 89-99.9%] in case the prior probability was 50% and 80% [95%CI 61-99%%] in case the prior probability was 20%. The threshold probability above which these findings should be accepted is 7% [0%-30%] if we assume B/H=13.5 or 41% [11-72%] if B/H=1.4. Table 1 summarizes the results.

The results indicate that in the best-case scenario the probability that research findings are true far exceeds the threshold above which the results should be accepted (i.e. PPV>p_t_). Therefore, rationally, in this case we should not hesitate to accept the findings from this study as truthful. However, in the worst case scenario lower limits of PPV’s 95% confidence intervals intersect with the upper limit of the threshold’s 95% confidence interval indicating that under these circumstances research hypothesis may not be acceptable (i.e. PPV is possibly < p_t_). Had the RTOG investigators made a mistake when they terminated the trial early? We now turn to this question.

**Dealing with unavoidable erroneous research findings**

Mistakes are integral part of research activities. We can always make a mistake and accept the results of research findings that are subsequently shown to be false(29). In due course, after making such a decision under uncertainty, we may discover that another alternative (i.e. the null hypothesis in our case) would have been preferable(12, 30-32). This knowledge may bring a sense of loss, or regret(30, 31, 33, 34). In fact, previous research indicated that in order to minimize anticipated regret, people may violate behavior as prescribed by classic expected utility decision-theory(33). Expected-utility theory is widely considered a cornerstone normative theory for rational decision-making(12, 13)(see also below and Appendix). However, abundant experience has also demonstrated that there are many situations in which we can tolerate wrong decisions, and others in which we cannot(2). We have previously described the concept of *acceptable regret* demonstrating that under certain conditions making a wrong [clinical] decision will not be particularly burdensome to the decision-maker (2). Here, we extend application of the concept of acceptable regret to the situation whether potentially false research findings should be tolerated.

**Defining tolerable limits for accepting potentially wrong research**

As indicated above, we can always make a wrong judgment and accept the findings from a research hypothesis when in fact it was false. Here, we are addressing the acceptance of research findings as a function of penalty (regret) for being wrong i.e. under which circumstances, if at all, we can accept a research findings that may subsequently shown to be false. *“Which decision (regarding research hypothesis) should we make if we want to ensure that the regret is less then a predetermined (minimal acceptable) regret, R_o_?”* That is, under these circumstances regret associated with wrongly accepting research hypothesis becomes *acceptable* (2).

As explained above, equation 2) was derived from application of classic decision- theory approach to the results of clinical trials stating that a rational decision-maker should select research vs. null-hypothesis depending on which one has the higher expected utility(3, 14-17). Extending the concept of acceptable regret within this framework, it can easily be shown (see Appendix) that we should be willing to accept results of potentially false research findings as long as probability (p) of it being true is above the threshold probability, p_r_ (equation 2):

$p\geq p_{r}=1-\frac{R_{0}}{H}$ 3)

R_0_ denotes acceptable regret and should be expressed in the same units as benefits and harms. Since regret among individuals do differ and is typically related to the magnitude of perceived benefits or harms(32, 35, 36), we will also assume the amount of acceptable regret is equal to the percentage of the benefits (r) that we are willing to lose in case our decision prove to be the wrong one.

Therefore, if $R_{o}=r\cdot B$ (the percentage of benefit), $1-\frac{R_{o}}{H}=1-r\cdot\frac{B}{H}$ 4)

Note, that there is nothing to prevents us from relating R_0_ to harms, or both benefits and harms. For example, if we express the amount of acceptable regret as a percentage (*s*) of harms that we are willing to tolerate even if our decision turns out to be the wrong one, $R_{o}=s\cdot H$ (a percentage of harms), formula (3) becomes:

$PPV\geq p_{r}=1-\frac{R_{o}}{H}=1-s\cdot\frac{H}{H}=1-s$ (4’)

These equations describe the effect of acceptable regret on the threshold probability (Eqs 1 and 2) in such a way that PPV now also needs to be above the thresholds defined in Eq. 3 [or Eq 4 (4’)] for research to become acceptable.

However, since most discussion is related to clinical research in which a typical research hypothesis is formulated to test for benefits, in this paper we focus on a relationship between *acceptable regret* and fraction of benefits that we are willing to forgo in the case of false research findings.

As indicated a rational decision-maker should select a research hypothesis only if it has a higher expected utility than the null-hypothesis(3, 14-17). However, when acceptable regret is taken into account, a decision-maker may choose to violate expected-utility precepts in order to minimize his sense of loss(2). Nevertheless, under some circumstances the threshold probability based on the classic decision-theoretic approach to research findings (equation 2) will be equal to the threshold probability derived from the acceptable regret approach. That is, these two thresholds will intersect at:

$\frac{1}{1+\frac{B}{H}}=1-r\cdot\frac{B}{H}$

Solving for benefit/harms ratio

$\frac{B}{H}=\frac{1}{r}-1$ 5)

and

$r\leq\frac{1}{1+\frac{B}{H}}$ 6)

Equation 6 indicated maximum possible loss (the largest value of r) that we can forgo (and still be wrong) while at the same time we adhere to the precepts of expected utility theory according to classic decision-theory. A practical interpretation of this inequality is that some research findings may never become acceptable unless we are ready to violate the axioms of expected utility theory i.e. accept value r to be larger than defined in equation 4 (Table 2).

**Example continued: acceptable regret and early stopping of clinical trials**

In this paper, we illustrate our approach using the example of the trial that evaluated the effects of Combined Rx vs. RT alone in patients with cancer of esophagus conducted by the RTOG investigators (27). Recall that, in our worst-case analysis (Table 1), we found that the probability that Combined Rx is better treatment could potentially be as low as 80% [61-99%] well overlapping with the probability of the threshold of 41% [11-72%]. Thus, it is quite conceivable that the investigators made a mistake when they closed the trial prematurely (since PPV could be less than p_t_; see Eq 1 and 2). If they wrongly terminated this study for presumed benefits, many future patients with cancer of esophagus would needlessly suffer and die because they would have been administered treatment that was subsequently found to be useless and harmful (25, 26). On the other hand, if they continued the trial, many patients would have been randomized to inferior RT arm as opposed to life-saving Combined Rx arm (25, 26).

We propose that one way to handle situations in which evidence is not solidly established is to explicitly take into the account a possibility that one can make a mistake and wrongly accept the results of research hypothesis, which subsequently may prove to be false. This, in turn, can help us determine “decision thresholds” that will take into account the amount of error which may or may not be particularly troublesome to us if we wrongly accept research findings. We contend that it could be very helpful if we make some assumptions about the extent of our tolerance for wrongly accepting the results of a research hypothesis.

Recall that the RTOG investigators hoped to detect the absolute difference between 10-30% of survival in favor of Combined Rx. By finding that Combined Rx improved survival by 29%, they appeared to have realized their most optimistic expectations(27). This implies that the investigators would consider their trial a success even if the survival was improved by 10% instead i.e. less than 67% of realized, but most optimistic outcome. Therefore, let’s assume that investigators in the esophagus cancer trial are prepared to accept that may be wrong and that were willing to forgo 10%, 30%, or 67% of benefits.

The calculated thresholds above which we should accept the findings from this study is in case of B/H=13.5 (best-case scenario), are zero regardless if assumed that our tolerable loss of benefits was 10%, 30%, or 67%, respectively. Note that these thresholds are well below calculated threshold that the hypothesis is true [95% (88-99.9%)] ( i.e. PPV > p_r_ = 0 for all acceptable regret assumptions; Eq 4) (Table 1).

However, in case of worst-case scenario, B/H=1.4, the calculated thresholds above which we should accept the findings from this study is 86% [95% CI: (84 – 88%)] for loss of 10% of benefits, 58% [95% CI: (52 – 64%)] for forgoing of 30% of net benefits and 6% [95% CI: (0 – 19%)] if we are willing to tolerate loss of 67% of net benefits (Fig 2). Note that except in the case when acceptable regret is 10% or less, probability that Combined Rx is better treatment [80% (61- 99%)] is above all other decision thresholds [because PPV (= 80% (61- 99%)) > acceptable regret threshold (=58% (52 – 64%)) and PPV > acceptable regret threshold (=6% (0 – 19%)] (see Eq 4) . Therefore, investigators seemed to have been correct when they terminated the trial earlier than originally anticipated. A long-term follow-up of this trial(37) and a meta-analysis of 7 randomized trials evaluating similar treatments(38) proved them subsequently right.

**Threshold probabilities for accepting findings in various types of clinical research**

Table 2 summarizes the results of most types of clinical research showing probabilities that research findings are true and above which may become acceptable for a given B/H ratio with and without acceptable regret being taken into account. What is remarkable is that depending on the amount of acceptable regret, our acceptance of potentially false research findings may dramatically change. For example, in case of a meta-analysis of small inconclusive studies we can accept its research hypothesis as true only if B/H>1.44. However, if we are willing to forgo, say, only 1% of net benefits in case we prove to be mistaken, the benefit/harm ratio for accepting the findings from the meta-analysis of small inconclusive studies dramatically increases to 59!

**Research results should be accepted when they are coherent with beliefs “upon which a man is prepared to act”**

Recently, Ioannidis (1) estimated that most published research findings are false, but he did not indicate when, if at all, potentially false research results may become acceptable. In the final analysis, the answer to the question posed in the title of this paper “when should potentially false research findings be accepted?” is an epistemological problem(39). We acknowledge that there is no a single objective probability of “truth”(40, 41); however, we contend that calculation of probability when research hypothesis is admissible is a doable exercise. We showed that acceptance of research hypothesis can be defined in terms of the degree of uncertainty (probabilities), research payback (benefits) and inadvertent consequences (harms) as well as associated error rates we are willing to tolerate when drawing conclusions as to whether the research findings under consideration are true or not.

We have employed a benefit-risk (harm) analysis based on reasoning that acceptance of research ultimately depends on its consequences- good and bad.

We should note here that despite its appeal and acceptance by researchers and regulators, such as the US Food and Drug Administration and the EPA (Environmental Protection Agency) , a concept of benefit-risk analysis remains paradoxically not clearly defined(42). For the most part, evaluation of benefits and harms is practiced as (non-simultaneous) qualitative exercise(42). However, to the extent that benefit-risk analysis represents a quantitative synthesis of favorable (benefits) and unfavorable (harms) effects of interventions that are being tested using a *common metrics* (ratio), the only possible analytical framework for performing such an analysis is to employ a decision-theory approach(3, 19, 42).

As intuitively expected, our analysis indicates that the greater the benefit/harm ratio, the less certain we need to be in the accuracy of research hypothesis to accept as true. When the signals are easily detectable (such as when B/H ratio is substantial) we are more inclined to accept the findings as true and self-evident even if the rigor of testing is not up to perfect standards (Table 2). Note, however, if the signal consists only of inadvertent consequences (harms) then we need to achieve the perfect certainty in order to accept research findings (Fig 1).

A reader should note that PPV i.e. evidence regarding the “truthfulness” of research results are expressed on the continuum scale from 0 to 100%. Ideally we should accept only those findings with PPV=100; however, in reality, as noted above, this ideal is not attainable. Acceptability of evidence should be looked at as a categorical, decision-making exercise in which we choose to accept research findings as “truthful” or not(43, 44). When degree of belief in the truthfulness of research hypothesis is sufficiently high, a rational person would choose to act upon it. Calculation of thresholds serves exactly this purpose. The threshold is a pivot around which we decide whether the hypothesis should be accepted or not: If the PPV is above threshold, then as indicated above, the results of research should be accepted.

As Ioannidis’s(1) and Wacholder et al.(6) our approach is based on Bayesian, inductive reasoning(11, 22) and acknowledges the impossibility of knowing the “absolute truth” using the calculus of inductive probability(40, 41). However, we are not calculating the probability of the “truth” but rather when research findings can be accepted as sufficiently valid. Although this may be less epistemologically pristine, at least in our opinion, it carries potentially enormous practical value since decisions have to be made. We follow the tradition advocating that the research hypothesis should be accepted when it is coherent with beliefs “upon which a man is prepared to act”(45).

We complemented the threshold with our *acceptable regret* approach(2) to show how *a priori* defining the limits of tolerance toward making mistakes can help us decide whether the research results should be accepted or not.

The answer to the question “when should we be ready to accept research findings” depends on the question of how much we are willing to tolerate that the research results may be wrong. Equation 4 shows an important result: if we are not willing to accept any loss of utility (regarding benefits) that our decision to accept research finding could be wrong (r=0), that would mean that we can operate only at absolute certainty in “truth” of research hypothesis (i.e. PPV=100%). Similarly, equation 4’ indicates that if we are not willing to tolerate any harms that may be associated with a wrong decision, we can live only with PPV=100%. This is clearly not attainable goal(1).

Therefore, our acceptability of “truth” depends how much we care about being wrong. The less we care; obviously the more relaxed is our definition of acceptability of research findings as true ones. If the tolerance toward error, our regret that we can be wrong and perceived benefits/harms associated with research is small, then we will generally require a higher probability that the findings are true. However, if we are willing to accept a greater possibility of erroneous conclusion in the results of research and perceived benefits/harms ratio is high, then the opposite conclusion may hold true: we may settle for less rigorous research results. Table 2 illustrates rather dramatic effects of acceptable regret on the calculation of the minimum benefit/harms thresholds above which research hypothesis can become acceptable.

A reader should note that we are not advocating here setting some arbitrary values that may contradict reasonable decisions by a sound decision-maker who (on his /her behalf or on behalf of society) has to make some value judgments. In fact, these values judgments occur on daily basis both at individual and policy-making levels. However, our value judgments about being wrong need to be balanced against our values toward benefit/harms. As noted above actions under expected utility theory and acceptable regret may not necessary be identical, but arguably the most rational course of action would be to select those research findings with the highest expected utility while keeping regret below the acceptable levels. This can be translated into a useful guide (Fig 3): the extent of acceptable regret as a function of benefit/harm ratio. It tells us that, within bounds of rational decision-making(12, 13, 46), there is some (rationally) acceptable amount of loss that we can afford to lose in case we are wrong. We mentioned above that sometimes expected utility decision-theory approach may conflict with acceptable regret approach if a decision-maker is a risk-averse and wants to keep his/her regret due to wrong decisions very low(2). Figure 3 (equation 6) shows the maximum percent of the net benefits that we may afford to lose as a function of benefit/harm ratio and still satisfy both requirements of rationality – the maximum expected utility and an acceptable level of regret. Shaded area in Table 2 indicates acceptability of research findings under acceptable regret conditions but which are in violation of expected utility theory(2). For example, in case of discovery-oriented exploratory research with massive testing ($\alpha=0.05,\beta=0.80$) according to expected utility threshold model we can accept its research hypothesis as true only if B/H>999. However, if we are willing to forgo, say, 20% of net benefits in case we prove to be mistaken, the benefit/harm ratio for accepting the findings from discovery-oriented exploratory research with massive testing dramatically drop to 5. Note, however, that our readiness to accept wrong decision violates the expected utility precepts of rational decision-making(2). People, of course, can choose to violate the precepts of expected utility theory-and in every-day decision-making they in fact commonly do (47). However, if that is done in the setting of scientific research, these reasons should be transparently and explicitly acknowledged and be shared with the public. Otherwise, theoretically, people can increase their level of acceptable regret in such a way that research findings can always become acceptable!

We believe that real usefulness of our methods will be derived if these values (benefit/harms that we are hoping to detect, acceptable regret) are decided in advance as researchers prespecify their false positive and false negative errors that they are ready to tolerate. This in turn would improve understanding of the entire scientific process and help us better understand a notion of “truth” and “falsity” in the research endeavor. By democratizing the process about the acceptable regret we believe that decisions regarding acceptability of research findings made by the research community and policy-makers, or other representatives of society at large, could be much improved.

**Limitations should be understood but more can be done**

Although we think that our method may be helpful under a variety of situations including decisions whether to terminate a clinical trial early, we need to acknowledge the limitations of our approach. Following Ioannidis, we have illustrated our methods using the example from clinical research, which also happened to be a field with which we are most familiar. However, our method can easily be generalized to other problems and fields although admittedly the terminology in terms of benefits and risks (harms) sounds rather awkward when employed in non-health care setting. Nevertheless, the principles of analyzing consequences (i.e. “gains” vs. “losses” in the broadest sense) associated with true or false research findings apply to all hypothesis-driven research. Perhaps, in non-clinical research employing a descriptive language would be more appropriate (e.g. differences in “true” and “false” outcomes under research and null hypothesis, respectively). This view, of course, is valid using a pragmatic approach to acceptability of scientific results, which is employed here. However, it can be argued that determining the acceptable limits of “truth” upon which we can act is not sole purpose of science. Therefore, the methods described here may not necessary be applicable to the fields where pure understanding is of only importance (i.e. without paying attention to the consequences of the result from a decision-maker perspective). Nevertheless, from economical development to environmental policies to clinical researches, the tradeoffs are an integral aspect of the scientific endeavors. In our attempts to balance these tradeoffs, human values toward benefits and harms and degree of errors that we can tolerate become essential.

Our method represents a necessary simplification in a very complex area of scientific inference. It focuses on a single hypothesis testing involving a single outcome. In reality, however, researchers, policy-makers and trialists often decide on the value of research hypothesis based on multiple endpoints, which is frequently combination of qualitative and quantitative reasoning. For example, when investigators decide whether to stop a clinical trial early, they may do it based not on rejection of the null-hypothesis for primary outcomes, but influenced by data on secondary outcomes, toxicities, compliance etc. Nevertheless, if decision is to be reached, it has been argued for some time that the focus should be on a single criterion-pragmatic approach although this criterion can be formed as a weighted combination of several single criteria based on investigators’ judgments about their importance(48). Therefore, given the fact that most of clinical research conducted today is driven by addressing a single hypothesis, we think that the method described here may be a very valuable complementary tool to further help operationalize a complex decision-making process. As discussed above, our model deals with a single-hypothesis evaluation and applies only to decision about a particular research hypothesis considered at a time and cannot extend beyond the specific question tested. A new question needs to be answered afresh for each particular research hypothesis. The ultimate validity of a specific claim should be made within the context of totality of research evidence and against a backlog of accepted scientific knowledge (49). This, however, applies to general scientific method as well and is not unique to our particular model.

It is also important to note that although the choice of benefit/harms measures is irrelevant in our model (e.g. life expectancy, morbidity or mortality rates, cost etc), decision-makers need to make sure that all variables (regret, benefit, harms) are expressed in the same units(2, 3). However, our model does not take simultaneously benefit/harms and costs in the same model. The problem is intimately linked to the question related to single vs. multi-attribute criteria discussed above and is not unique to our model. A solution has not been easy because involve the value judgments, but within consistency of decision-makers’ goals and values, we think it is possible to integrate costs with other [clinical] research outcomes in the same model. This will be subject of the future work.

Finally, this paper highlights, from a somewhat different perspective, the importance of knowing benefits and harms to make informed (scientific) decisions. As evidence-based medicine movement has repeatedly stressed over the last 15 years in the context of clinical management, physicians and patients cannot make informed decisions if reliable data on treatments about benefits and harms are not collected or reported. We now show that this is essential for informed scientific decisions as well. We call for explicit reporting of consequences (“good” and “bad”) related to the outcomes observed in scientific research.

It should be, however, noted that because a typical clinical research hypothesis is formulated to test for benefits, we have here postulated a relationship between *acceptable regret* and fraction of benefits that we are willing to forgo in the case of false research findings. Unfortunately, when we move outside the realm of medical treatments and interventions, harms and benefits are very difficult to quantify and envision in all their immediate and long-term consequences. Occasionally, wrong adoption of some false positive findings may lead to the adoption of other false findings thus creating fields replete with spurious claims. Even for clinical medicine, where benefits and harms are more typically measured, we should acknowledge that often the quality of the information on harms is suboptimal (50). There is no guarantee that the "benefits" will exceed the "harms". Although as noted above there is nothing to prevents us from relating R_0_ to harms, or both benefits and harms, one must acknowledge there is much more uncertainty, often total ignorance, about the harms data. As a consequence, under these circumstances research may become acceptable only if we relax acceptability regret criteria i.e. accept value r to be larger than defined in equation 6. That is, unless we are ready to violate the precepts of rational decision-making (shaded area in Table 2) a research finding with low PPV (the majority of research findings) should not be accepted(1). [Theoretically, there is always a value of r and B/H ratio-however, unrealistic-at which the expected utility theory will agree with acceptable regret criteria. For example, to accept discovery-oriented exploratory research with massive testing (PPV=0.10%), Ro will have to be smaller than 0.10% benefits, and B/H ratio has to be at least 999. None of these values appear to be practically sensible choices.]

We conclude that since obtaining absolute “truth” in research is impossible, society has to decide when less-than-perfect results may become acceptable. The approach presented here may facilitate the decision-making in scientific research.

| **Table 1. How true is the research hypothesis that combined chemotherapy is superior to radiotherapy alone in the management of cancer of esophagus?*** | | | | | | |
| --- | --- | --- | --- | --- | --- | --- |
| **Net benefits**  **(survival; %)%** | **Net Harms**  **[treatment-related mortality; %]%** | **Benefit/harms ratio** | **Type I (α) error** | **Type II (β) error** | **The threshold probability above which research hypothesis should be accepted as true findings (%)** | **Probability that research hypothesis is true (%)** |
| [88-59-2]** =27% | 2% | 13.5 | 5% | 10% | 7% [0%-30%] | 95%  [89-99.9%]^$^ |
| [88-59-12]*** =17% | 12% | 1.4 | 5% | 20% | 41% [11-72%] | 80% ^#^  [61-99%] |

%- calculated as described in reference (19); *from reference(27); **- best-case scenario; ***-worst-case scenario; ^$-^assumes 50% prior probability; ^#^- assumes 20% prior probability

| **Table 2 Probability that research findings are true and above which may become acceptable** | | | | |
| --- | --- | --- | --- | --- |
| **Type of research** | **Probability that findings are true*** | **Minimum benefit/harms ratio above which research hypothesis can be acceptable**  **(no regret taken into account)** | **Acceptable regret** for wrongly accepting research hypothesis** | **Minimum benefit/harms ratio above which alternative hypothesis can be acceptable**  **(when acceptable regret** is taken into account)** |
| Adequately powered RCT with little bias and 1:1: pre-study odds (*β* = 20%) | 85% | 0.18 | 1% | 15 |
|  |  |  | 20% | 0.75 |
|  |  |  | 30% | 0.50 |
|  |  |  | 40% | 0.38 |
|  |  |  | 60% | 0.25 |
|  |  |  | 80% | 0.19 |
| Confirmatory meta-analysis of good quality RCTs (*β* = 5%) | 85% | 0.18 | 1% | 15 |
|  |  |  | 20% | 0.75 |
|  |  |  | 30% | 0.50 |
|  |  |  | 40% | 0.38 |
|  |  |  | 60% | 0.25 |
|  |  |  | 80% | 0.19 |
| Meta-analysis of small inconclusive studies (*β* = 20%) | 41% | 1.44 | 1% | 59 |
|  |  |  | 20% | 2.95 |
|  |  |  | 30% | 1.97 |
|  |  |  | 40% | 1.48 |
|  |  |  | 60% | 0.98 |
|  |  |  | 80% | 0.74 |
| Underpowered, but well-performed phase I/II RCT (*β* = 80%) | 23% | 3.35 | 1% | 77 |
|  |  |  | 20% | 3.85 |
|  |  |  | 30% | 2.57 |
|  |  |  | 40% | 1.93 |
|  |  |  | 60% | 1.28 |
|  |  |  | 80% | 0.96 |
| Underpowered, poorly performed phase I/II RCT (*β* = 80%) | 17% | 4.88 | 1% | 83 |
|  |  |  | 20% | 4.15 |
|  |  |  | 30% | 2.77 |
|  |  |  | 40% | 2.08 |
|  |  |  | 60% | 1.38 |
|  |  |  | 80% | 1.04 |
| Adequately powered exploratory epidemiological study (*β* = 20%) | 20% | 4.00 | 1% | 80 |
|  |  |  | 20% | 4 |
|  |  |  | 30% | 2.67 |
|  |  |  | 40% | 2 |
|  |  |  | 60% | 1.33 |
|  |  |  | 80% | 1 |
| Underpowered exploratory epidemiological study (*β* = 80%) | 12% | 7.33 | 1% | 88 |
|  |  |  | 20% | 4.4 |
|  |  |  | 30% | 2.93 |
|  |  |  | 40% | 2.2 |
|  |  |  | 60% | 1.47 |
|  |  |  | 80% | 1.1 |
| Discovery-oriented exploratory research with massive testing (*β* = 80%) | 0.10% | 999.00 | 1% | 99 |
|  |  |  | 20% | 5 |
|  |  |  | 30% | 3.33 |
|  |  |  | 40% | 2.5 |
|  |  |  | 60% | 1.67 |
|  |  |  | 80% | 1.25 |
| As in previous example, but with more limited bias (more standardized) (*β* = 80%) | 0.15% | 665.67 | 1% | 99.85 |
|  |  |  | 20% | 4.99 |
|  |  |  | 30% | 3.33 |
|  |  |  | 40% | 2.5 |
|  |  |  | 60% | 1.66 |
|  |  |  | 80% | 1.25 |

*-data from Ioannidis, 2005 **-expressed as r=1%,10%,20% of benefits (i.e. percentage of benefits that we can tolerate losing in case we wrongly accept research findings); Shaded area: applies only if a decision-maker is willing to violate precepts of rational decision-making under expected utility of theory; otherwise under these circumstances research hypothesis **never** becomes acceptable [research may become acceptable if regret is smaller than the values prespecified in the table; see text (equation 6)]

Fig 1. The threshold probability **above** which we should accept findings of research hypothesis as being true. The horizontal yellow line indicates the actual conditional probability that the research hypothesis is true in the case of positive findings. This means that for benefit/harms (B/H) ratio above the threshold (1.5 in this example) the research hypothesis can be accepted.

Fig 2.

The threshold probability (*P_t_*) **above** which we should accept findings of research hypothesis as being true (pink line) as a function of benefit/harm (B/H) ratio. Three calculated (acceptable regret) thresholds above which we should accept research findings are shown for the worst case scenario (B/H=1.4- see text for details) as a function of willingness to forgo benefits of 10%, 30% or 67% (slanted line). This means that as long as probability that research findings are above the acceptable regret thresholds (horizontal lines) they could be accepted without regret in case research hypothesis proves to be wrong (see text for details and Fig 2 of the shorter version of the paper).

Figure 3. The curves show the extent the amount of benefits we can afford to lose (acceptable regret) as a function of benefit/harms ratio in case we are wrong in accepting research hypothesis (see text for details)

# Appendix

### Analogies between Hypothesis Testing and Clinical Management

In a typical **Hypothesis Testing** scenario we have to accept one of the two hypotheses, the null hypothesis (*H_o_*) and the alternative, research hypothesis (*H_a_*). The possible results of our testing are two research findings: *RF+* = “research finding positive”, and *RF-* = “research finding negative”. The true state of reality can be described as either *H_a_-* = “null hypothesis is true (research hypothesis is false)”, and *H_a_+* = “null hypothesis is false (research hypothesis is true)”.

The probability of false negative research finding (type II error) is $\beta=P\left(RF-|H_{a}+\right)$ and is usually set at $\beta=0.20$. The probability of a false positive research finding (type I error) is $\alpha=P\left(RF+|H_{a}-\right)$; typically *α* is assumed to be$\alpha=0.05$. The positive and negative likelihood ratios are $LR+=\frac{1-\beta}{\alpha}$ and $LR-=\frac{\beta}{1-\alpha}$.

In a typical **Clinical Testing** scenario we conduct a clinical test in order to confirm one of the two hypotheses, the absence of disease (*H_o_*) or the alternative, the presence of the disease (*H_a_*). The possible results of our testing are two test results: *RF+* = “positive test result” (indicates presence of the disease), and *RF-* = “negative test result”. The true state of reality can be described as either *H_a_-* = “the patient does not have the disease.”, and *H_a_+* = “the patient has the disease”.

The probability of false negative test result is $\beta=P\left(RF-|H_{a}+\right)$ (the opposite of sensitivity, $\beta=1-S$). The probability of a false positive test result is $\alpha=P\left(RF+|H_{a}-\right)$ (the opposite of specificity, $\alpha=1-S_{p}$). The positive and negative likelihood ratios are $LR+=\frac{S}{1-S_{p}}=\frac{1-\beta}{\alpha}$ and $LR-=\frac{1-S}{S_{p}}=\frac{\beta}{1-\alpha}$.

The posterior probability that the positive research finding (presence of the disease) is accurate is given by $PPV=P\left(H_{a}+|RF+\right)=\frac{P\left(RF+|H_{a}+\right)P\left(H_{a}+\right)}{P\left(RF+|H_{a}+\right)P\left(H_{a}+\right)+P\left(RF+|H_{a}-\right)P\left(H_{a}-\right)}$.

If we denote by $\pi=P\left(H_{a}+\right)$ the prior probability of positive research finding (presence of the disease), we can rewrite the equation above as $PPV=\frac{\left(1-\beta\right)\pi}{\left(1-\beta\right)\pi+\alpha\left(1-\pi\right)}$.

### Decision Theory and Expected Utilities

Net benefit, B, is the difference between the utilities of outcomes of the action taken under research hypothesis and the null hypothesis, respectively (when in fact research hypothesis is true), $B=U_{1}-U_{3}$ (see Figure A.1). Net harms, H, are defined as the difference between the utilities of outcomes of the action taken under the null and research hypothesis, respectively (when in fact null hypothesis is true), $H=U_{4}-U_{2}$ (see Figure A.1).

In the context of classical decision theory (see Figure A1), we select the hypothesis with higher expected utility (EU) involving Benefits and Harms associated with our decision. Expected utility is the average of all possible results weighted by their corresponding probabilities. In case of the positive Research Finding $\left(RF+\right)$, the expected utility of accepting the alternate hypothesis is $E\left(H_{a}\right)=PPV\cdot U_{1}+\left(1-PPV\right)\cdot U_{2}$, and the expected utility of accepting the null hypothesis is $E\left(H_{0}\right)=PPV\cdot U_{3}+\left(1-PPV\right)\cdot U_{4}$. Setting $\text{E}\left(\text{H}_{\text{a}}\right)\text{=E}\left(\text{H}_{\text{o}}\right)$and solving for p, we find the probability at which either decision results in the same expected utility, the threshold probability (*p_t_*):

$p_{t}=\frac{1}{1+\left(\frac{B}{H}\right)}$ (A.1)

In terms of prior probability, *π* (the probability that the research hypothesis is true before we perform the hypothesis testing), this threshold splits into two threshold probabilities: a) the treatment threshold probability above which we will accept the results of research hypothesis (reject a null hypothesis) (*p_tRH_*) and b) the no-treatment threshold probability below which we will accept the results of null hypothesis (*p_tNH_*). Mathematically these two thresholds can be expressed as:

$p_{t}=\frac{1}{1+LR\cdot\frac{B}{H}}$ (A.1’)
where *p_t_* = *p_tRH_* if , and *p_t_* = *p_tNH_* if.

The minimum B/H ratio for the given posterior probability for which the research hypothesis has a greater expected utility than the null hypothesis will occur when:

$\frac{1}{1+\left(\frac{B}{H}\right)}\leq PPV$ or $\frac{1}{PPV}-1=\frac{1-PPV}{PPV}\leq\left(\frac{B}{H}\right)$ (A.2)
In terms of prior probability *π*, we can rewrite the formula (2) as

$\frac{1-\pi}{\pi}\cdot\frac{1}{\left(LR-\right)}\leq\left(\frac{B}{H}\right)$ (A.2’)

### Regret and Acceptable Regret

A reader is referred to reference (2) for details. Briefly, regret (Rg) is the difference between the utility of the outcome of the action taken and the utility of the outcome of another action we should have taken, in retrospect. For example, regret associated with acceptance of the research hypothesis (treatment Rx1) when in fact the null hypothesis is true is given by

$$\text{Rg(Rx1,H}_{\text{a}}\text{-) = max[U(Rx1,H}_{\text{a}}\text{-), U(Rx2,H}_{\text{a}}\text{-)] - U(Rx1,H}_{\text{a}}\text{-) = U}_{4}\text{-U}_{2}\text{= H}$$

The regret associated with acceptance of the null hypothesis (treatment Rx2) when in fact the null hypothesis is true is given by

$$\text{Rg(Rx2,H}_{\text{a}}\text{-) = max[U(Rx1,H}_{\text{a}}\text{-), U(Rx2,H}_{\text{a}}\text{-)] - U(Rx2,H}_{\text{a}}\text{-) = U}_{4}\text{-U}_{4}\text{= 0}$$

The regret associated with acceptance of the null hypothesis (treatment Rx2) when in fact the research hypothesis is true is given by

$$\text{Rg(Rx2,H}_{\text{a}}\text{+) = max[U(Rx1,H}_{\text{a}}\text{+), U(Rx2,H}_{\text{a}}\text{+)] - U(Rx2,H}_{\text{a}}\text{+) = U}_{1}\text{-U}_{3}\text{= B}$$

The regret associated with acceptance of the research hypothesis (treatment Rx1) when in fact the research hypothesis is true is given by

$$\text{Rg(Rx1,H}_{\text{a}}\text{+) = max[U(Rx1,H}_{\text{a}}\text{+), U(Rx2,H}_{\text{a}}\text{+)] - U(Rx1,H}_{\text{a}}\text{+) = U}_{1}\text{-U}_{1}\text{= 0}$$

Repeating the expected utilities procedure described above, we can define expected regret associated with selection of the research and the null hypothesis, respectively. A solution of these two equations will produce the same equation (A1) as the one defined under classic expected utility theory. However, at the intersection where the decisions of selecting the research hypothesis vs. the null are the same, the expected regret ($ER\left[.\right]$) is maximal:

$$ER\left[Rx1\right]=\left(1-p_{t}\right)H=ER\left[Rx2\right]=p_{t}\cdot B=\frac{B}{1+\frac{B}{H}}$$

Unlike the threshold probability, the maximal level of expected regret does not depend on the benefit/harm ratio only but also on the absolute magnitude of the net benefit.

The acceptable regret, *R_0_*, is the utility we find acceptable of losing. We are interested in finding out at which probability *ER[Rx1]≤ R_0_.* Solving this inequality, it follows that we should be willing to accept results of potentially false research findings as long as probability (p) of it being true is above the threshold probability, p_r_

$p\geq p_{r}=1-\frac{R_{0}}{H}$ (A.3)

Since regret among individuals does differ and is typically related to the magnitude of perceived benefits or harms, we will also assume the amount of acceptable regret is equal to the percentage of the benefits (*r*% of benefits) that we are willing to lose in case our decision proves to be the wrong one.

Therefore, if $R_{o}=r\cdot B$ (the percentage of benefit),

$p\geq p_{r}=1-\frac{R_{o}}{H}=1-r\cdot\frac{B}{H}$ (A.4)

When acceptable regret is taken into account, a decision-maker may choose to violate expected-utility precepts in order to minimize his sense of loss. For example, if the probability PPV is between the thresholds $\frac{1}{1+\frac{B}{H}}\leq PPV\leq1-r\cdot\frac{B}{H}$, the expected utility theory would prescribe acceptance of the research hypothesis (Rx1), but the decision maker would be exposed to an uncomfortable level of expected regret.

Nevertheless, under some circumstances the threshold probability based on the classic decision-theoretic approach to research findings (equation A.1) will be equal to the threshold probability derived from the acceptable regret approach. That is, these two thresholds will intersect when: $\frac{1}{1+\frac{B}{H}}=1-r\cdot\frac{B}{H}$.  Solving for benefit/harms ratio, we have: $\frac{B}{H}=\frac{1}{r}-1$ (A.5)

or, solving for *r*: $r=\frac{1}{1+\frac{B}{H}}$ (A.6)

Equation A.6 indicated maximum possible loss (as a percent of the benefit) that we are willing to forgo (and be wrong) while at the same time adhering to the precepts of expected utility theory according to classic decision-theory.

### Confidence intervals

Using Taylor’s expansion we can approximate the variance of a multivariable function of independent variables $w=f\left(x_{1},x_{2},x_{3},...,x_{k}\right)$ using the formula $\sigma_{w}^{2}=\sum_{i=1}^{k} \left(\frac{\partial f\left(x_{1},x_{2},x_{3},...,x_{k}\right)}{\partial x_{i}}\right)^{2}\sigma_{x_{i}}^{2}$. Using this formula we can estimate the confidence intervals for the variables in formulas (A.1) – (A.6). For details, see the reference (7).

**
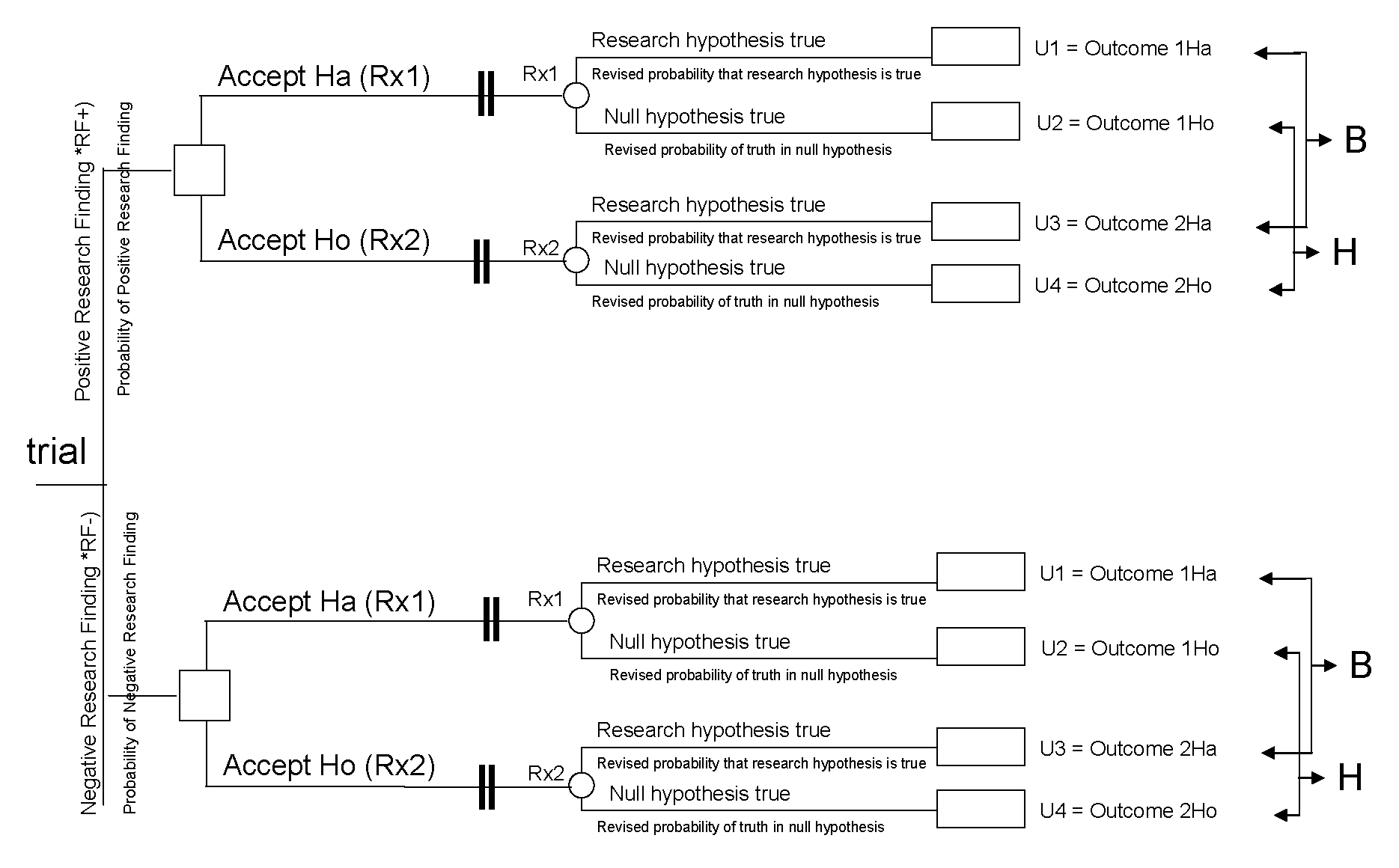
**

**Figure A1:** Decision tree outlining the choice in a typical clinical research setting between accepting research hypothesis (H_a_: Treatment Rx1 is superior) vs. null hypothesis (H_o_: Rx2 is superior).

**References:**
