# Supplementary material for: When Should Potentially False Research Findings Be Considered Acceptable?
Source: PLoS Med. 2007 Feb 27;4(2):e26. doi: 10.1371/journal.pmed.0040026 (PMC1808081; doi:10.1371/journal.pmed.0040026)
Supplement: Text S2 — (163 KB DOC). [file pmed.0040026.sd002.doc]

# Appendix: When should potentially false research findings be acceptable?

Benjamin Djulbegovic and Iztok Hozo

(see PLoS Medicine 2007;4(2):e26.doi:10.1371/journal.pmed.0040026)

### Analogies between Hypothesis Testing and Clinical Management

In a typical **Hypothesis Testing** scenario we have to accept one of the two hypotheses, the null hypothesis (*H_o_*) and the alternative, research hypothesis (*H_a_*). The possible results of our testing are two research findings: *RF+* = “research finding positive”, and *RF-* = “research finding negative”. The true state of reality can be described as either *H_a_-* = “null hypothesis is true (research hypothesis is false)”, and *H_a_+* = “null hypothesis is false (research hypothesis is true)”.

The probability of false negative research finding (type II error) is $\beta=P\left(RF-|H_{a}+\right)$ and is usually set at $\beta=0.20$. The probability of a false positive research finding (type I error) is $\alpha=P\left(RF+|H_{a}-\right)$; typically *α* is assumed to be$\alpha=0.05$. The positive and negative likelihood ratios are $LR+=\frac{1-\beta}{\alpha}$ and $LR-=\frac{\beta}{1-\alpha}$.

In a typical **Clinical Testing** scenario we conduct a clinical test in order to confirm one of the two hypotheses, the absence of disease (*H_o_*) or the alternative, the presence of the disease (*H_a_*). The possible results of our testing are two test results: *RF+* = “positive test result” (indicates presence of the disease), and *RF-* = “negative test result”. The true state of reality can be described as either *H_a_-* = “the patient does not have the disease.”, and *H_a_+* = “the patient has the disease”.

The probability of false negative test result is $\beta=P\left(RF-|H_{a}+\right)$ (the opposite of sensitivity, $\beta=1-S$). The probability of a false positive test result is $\alpha=P\left(RF+|H_{a}-\right)$ (the opposite of specificity, $\alpha=1-S_{p}$). The positive and negative likelihood ratios are $LR+=\frac{S}{1-S_{p}}=\frac{1-\beta}{\alpha}$ and $LR-=\frac{1-S}{S_{p}}=\frac{\beta}{1-\alpha}$.

The posterior probability that the positive research finding (presence of the disease) is accurate is given by $PPV=P\left(H_{a}+|RF+\right)=\frac{P\left(RF+|H_{a}+\right)P\left(H_{a}+\right)}{P\left(RF+|H_{a}+\right)P\left(H_{a}+\right)+P\left(RF+|H_{a}-\right)P\left(H_{a}-\right)}$.

If we denote by $\pi=P\left(H_{a}+\right)$ the prior probability of positive research finding (presence of the disease), we can rewrite the equation above as $PPV=\frac{\left(1-\beta\right)\pi}{\left(1-\beta\right)\pi+\alpha\left(1-\pi\right)}$.

Interested readers can found further details in the references(1-4).

### Decision Theory and Expected Utilities

Net benefit, B, is the difference between the utilities of outcomes of the action taken under research hypothesis and the null hypothesis, respectively (when in fact research hypothesis is true), $B=U_{1}-U_{3}$ (see Figure A.1). Net harms, H, are defined as the difference between the utilities of outcomes of the action taken under the null and research hypothesis, respectively (when in fact null hypothesis is true), $H=U_{4}-U_{2}$ (see Figure A.1).

In the context of classical decision theory (see Figure A1), we select the hypothesis with higher expected utility (EU) involving Benefits and Harms associated with our decision. Expected utility is the average of all possible results weighted by their corresponding probabilities. In case of the positive Research Finding $\left(RF+\right)$, the expected utility of accepting the alternate hypothesis is $E\left(H_{a}\right)=PPV\cdot U_{1}+\left(1-PPV\right)\cdot U_{2}$, and the expected utility of accepting the null hypothesis is $E\left(H_{0}\right)=PPV\cdot U_{3}+\left(1-PPV\right)\cdot U_{4}$. Setting $\text{E}\left(\text{H}_{\text{a}}\right)\text{=E}\left(\text{H}_{\text{o}}\right)$and solving for p, we find the probability at which either decision results in the same expected utility, the threshold probability (*p_t_*)(5):

$p_{t}=\frac{1}{1+\left(\frac{B}{H}\right)}$ (A.1)

In terms of prior probability, *π* (the probability that the research hypothesis is true before we perform the hypothesis testing), this threshold splits into two threshold probabilities: a) the treatment threshold probability above which we will accept the results of research hypothesis (reject a null hypothesis) (*p_tRH_*) and b) the no-treatment threshold probability below which we will accept the results of null hypothesis (*p_tNH_*). For unbiased results, mathematically these two thresholds can be expressed as(6, 7):

$p_{t}=\frac{1}{1+LR\cdot\frac{B}{H}}$ (A.1’)
where *p_t_* = *p_tRH_* if , and *p_t_* = *p_tNH_* if.

Ioannidis(8) defines the effect of bias (u) on research findings in such a way that a fraction of false negative results can be classified as true positives (u*β) and a fraction of true negatives can be classified as false positives [u*(1-α)] because of bias. When incorporating bias, expressions for LR become(9):

$LR+=\frac{[(1-\beta)+u*\beta]}{[\alpha+u*(1-\alpha)]}=\frac{[(1-\beta)*(1-u)]}{[\alpha*(1-u)+u]}$ and $LR-=\frac{[\beta*(1-u)]}{[(1-\alpha)*(1-u)]}$

Note that under this definition, bias effects cancel out in the expression for LR-. This indicate that it would be more desirable to assume separate effects of bias on true positive results (u1) and true negative results (u2), respectively.

The minimum B/H ratio for the given posterior probability for which the research hypothesis has a greater expected utility than the null hypothesis will occur when:

$\frac{1}{1+\left(\frac{B}{H}\right)}\leq PPV$ or $\frac{1}{PPV}-1=\frac{1-PPV}{PPV}\leq\left(\frac{B}{H}\right)$ (A.2)
In terms of prior probability *π*, we can rewrite the formula (2) as

$\frac{1-\pi}{\pi}\cdot\frac{1}{\left(LR-\right)}\leq\left(\frac{B}{H}\right)$ (A.2’)

### Regret and Acceptable Regret

A reader is referred to reference (10) for details. Briefly, regret (Rg) is the difference between the utility of the outcome of the action taken and the utility of the outcome of another action we should have taken, in retrospect. For example, regret associated with acceptance of the research hypothesis (treatment Rx1) when in fact the null hypothesis is true is given by

$$\text{Rg(Rx1,H}_{\text{a}}\text{-) = max[U(Rx1,H}_{\text{a}}\text{-), U(Rx2,H}_{\text{a}}\text{-)] - U(Rx1,H}_{\text{a}}\text{-) = U}_{4}\text{-U}_{2}\text{= H}$$

The regret associated with acceptance of the null hypothesis (treatment Rx2) when in fact the null hypothesis is true is given by

$$\text{Rg(Rx2,H}_{\text{a}}\text{-) = max[U(Rx1,H}_{\text{a}}\text{-), U(Rx2,H}_{\text{a}}\text{-)] - U(Rx2,H}_{\text{a}}\text{-) = U}_{4}\text{-U}_{4}\text{= 0}$$

The regret associated with acceptance of the null hypothesis (treatment Rx2) when in fact the research hypothesis is true is given by

$$\text{Rg(Rx2,H}_{\text{a}}\text{+) = max[U(Rx1,H}_{\text{a}}\text{+), U(Rx2,H}_{\text{a}}\text{+)] - U(Rx2,H}_{\text{a}}\text{+) = U}_{1}\text{-U}_{3}\text{= B}$$

The regret associated with acceptance of the research hypothesis (treatment Rx1) when in fact the research hypothesis is true is given by

$$\text{Rg(Rx1,H}_{\text{a}}\text{+) = max[U(Rx1,H}_{\text{a}}\text{+), U(Rx2,H}_{\text{a}}\text{+)] - U(Rx1,H}_{\text{a}}\text{+) = U}_{1}\text{-U}_{1}\text{= 0}$$

Repeating the expected utilities procedure described above, we can define expected regret associated with selection of the research and the null hypothesis, respectively. A solution of these two equations will produce the same equation (A1) as the one defined under classic expected utility theory. However, at the intersection where the decisions of selecting the research hypothesis vs. the null are the same, the expected regret ($ER\left[.\right]$) is maximal:

$$ER\left[Rx1\right]=\left(1-p_{t}\right)H=ER\left[Rx2\right]=p_{t}\cdot B=\frac{B}{1+\frac{B}{H}}$$

Unlike the threshold probability, the maximal level of expected regret does not depend on the benefit/harm ratio only but also on the absolute magnitude of the net benefit.

The acceptable regret, *R_0_*, is the utility we find acceptable of losing. We are interested in finding out at which probability *ER[Rx1]≤ R_0_.* Solving this inequality, it follows that we should be willing to accept results of potentially false research findings as long as probability (p) of it being true is above the threshold probability, p_r_

$p\geq p_{r}=1-\frac{R_{0}}{H}$ (A.3)

Since regret among individuals does differ and is typically related to the magnitude of perceived benefits or harms, we will also assume the amount of acceptable regret is equal to the percentage of the benefits (*r*% of benefits) that we are willing to lose in case our decision proves to be the wrong one.

Therefore, if $R_{o}=r\cdot B$ (the percentage of benefit),

$p\geq p_{r}=1-\frac{R_{o}}{H}=1-r\cdot\frac{B}{H}$ (A.4)

When acceptable regret is taken into account, a decision-maker may choose to violate expected-utility precepts in order to minimize his sense of loss. For example, if the probability PPV is between the thresholds $\frac{1}{1+\frac{B}{H}}\leq PPV\leq1-r\cdot\frac{B}{H}$, the expected utility theory would prescribe acceptance of the research hypothesis (Rx1), but the decision maker would be exposed to an uncomfortable level of expected regret.

Nevertheless, under some circumstances the threshold probability based on the classic decision-theoretic approach to research findings (equation A.1) will be equal to the threshold probability derived from the acceptable regret approach. That is, these two thresholds will intersect when: $\frac{1}{1+\frac{B}{H}}=1-r\cdot\frac{B}{H}$.  Solving for benefit/harms ratio, we have: $\frac{B}{H}=\frac{1}{r}-1$ (A.5)

or, solving for *r*: $r=\frac{1}{1+\frac{B}{H}}$ (A.6)

Equation A.6 indicated maximum possible loss (as a percent of the benefit) that we are willing to forgo (and be wrong) while at the same time adhering to the precepts of expected utility theory according to classic decision-theory.

### Confidence intervals

Using Taylor’s expansion we can approximate the variance of a multivariable function of independent variables $w=f\left(x_{1},x_{2},x_{3},...,x_{k}\right)$ using the formula $\sigma_{w}^{2}=\sum_{i=1}^{k} \left(\frac{\partial f\left(x_{1},x_{2},x_{3},...,x_{k}\right)}{\partial x_{i}}\right)^{2}\sigma_{x_{i}}^{2}$. Using this formula we can estimate the confidence intervals for the variables in formulas (A.1) – (A.6). For details, see the reference(11).

**
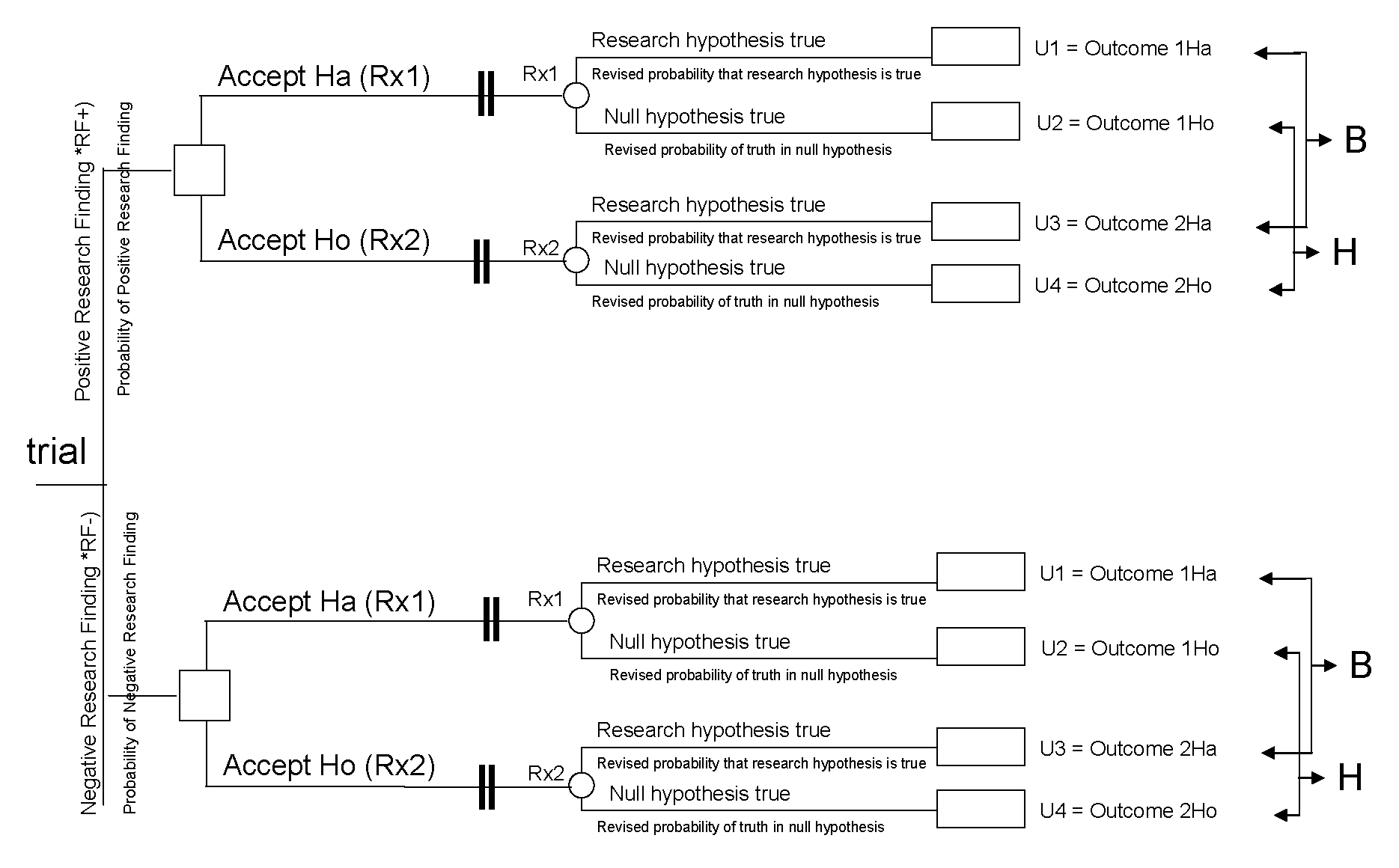
**

**Figure A1:** Decision tree outlining the choice in a typical clinical research setting between accepting research hypothesis (H_a_: Treatment Rx1 is superior) vs. null hypothesis (H_o_: Rx2 is superior).

**References:**

1. Browner WS, Newman TB. Are all significant p values created equal. The analogy between diagnostic tests and clinical research. JAMA. 1987;257:2459-63.

2. Goodman SN. Toward evidence-based medical statitistics. 1: the p value fallacy. Ann Intern Med. 1999;130:995-1004.

3. Goodman SN. Toward evidence-based medical statistics. 2: the Bayes factor. Ann Intern Med. 1999;130:1005-13.

4. Pianadosi S. Clinical Trials. A Methodologic perspective. Hoboken, New Jersey: Wiley-Interscience; 2005.

5. Pauker S, Kassirer J. Therapeutic decision making: a cost benefit analysis. N Engl J Med. 1975;293:229 -34.

6. Djulbegovic B, Desoky AH. Equation and nomogram for calculation of testing and treatment thresholds. Med Decis Making. 1996 Apr-Jun;16(2):198-9.

7. Djulbegovic B, Hozo I. At what degree of belief in a research hypothesis is a trial in humans justified? J Eval Clin Practice. 2002;8:269-76.

8. Ioannidis JP. Why most published research findings are false. PLoS Med. 2005 Aug;2(8):e124.

9. Pauker SG. The clinical interpretation of research. PLoS Medicine. 2005;2:e395.

10. Djulbegovic B, Hozo I, Schwartz A, McMasters K. Acceptable regret in medical decision making. Med Hypotheses. 1999;53:253-9.

11. Hozo I, Djulbegovic B. Calculating confidence intervals for posttest and threshold probabilities. MD Computing. 1997;15:110-5.
